# Artificial Intelligence, Machine Learning, and Deep Learning Approaches for Attention-Deficit/Hyperactivity Disorder Diagnosis: Protocol for a Scoping Review

**DOI:** 10.2196/99551

**Published:** 2026-10-07

**Authors:** Benedict Onochie Ibe, Amir Aly, Shaymaa S Al-Juboori, Emmanuel Ifeachor, Rohit Shankar

**Affiliations:** 1 School of Engineering, Computing and Mathematics, University of Plymouth, Drake Circus, Plymouth, Devon, England, PL4 8AA, United Kingdom, 44 07476836637; 2 Cornwall Intellectual Disability Equitable Research (CIDER), Plymouth, Devon, United Kingdom

**Keywords:** attention-deficit/hyperactivity disorder, ADHD, artificial intelligence, AI, machine learning, deep learning, explainable AI, scoping review, diagnostic classification

## Abstract

**Background:**

Attention-deficit/hyperactivity disorder (ADHD) affects over 366 million adults and 139 million children worldwide, yet diagnosis remains fundamentally subjective, relying on clinical interviews, behavioral observations, and rating scales that yield inconsistent results across practitioners and settings. Artificial intelligence (AI), machine learning (ML), and deep learning (DL) offer a paradigm shift toward objective, data-driven diagnosis by detecting complex patterns across neuroimaging, electrophysiology, and digital biomarkers that elude conventional assessment. Although AI-based ADHD research has grown exponentially, no comprehensive synthesis examines the full spectrum of data modalities, validation practices, and clinical translation readiness. This gap limits our understanding of which approaches are most promising for real-world implementation.

**Objective:**

This scoping review will visually map the current evidence on AI-based ADHD classification with respect to predictive accuracy, data forms, data features, generalizability, and interpretability of models.

**Methods:**

This scoping review will use the Joanna Briggs Institute approach to scoping reviews and follow the PRISMA-ScR (Preferred Reporting Items for Systematic Reviews and Meta-Analyses extension for Scoping Reviews) guidelines. Five electronic databases (IEEE Xplore, Scopus, PubMed, Web of Science, and ACM Digital Library) will be systematically searched for peer-reviewed studies published between January 2019 and April 2026. Empirical studies in English involving the use of AI, ML, DL, or explainable AI to diagnose ADHD with a total sample size greater than 100 participants (ADHD and control groups combined) and a non-ADHD control group (individuals with typical development or healthy individuals) will be included. Two independent reviewers will screen the titles, abstracts, and full texts, and any conflicts will be resolved through either discussion or arbitration. A standardized Microsoft Excel template will be used to extract data that will include the following: bibliographic data, data modalities, data models, data validation approaches, performance metrics, and explainability approaches. Thematic and narrative analysis will be used to synthesize findings on 4 research questions that will address model performance, data modality contributions, data characteristics, and interpretability methods.

**Results:**

The scoping review began in December 2025. Analysis and screening are in progress, with the scoping review expected to be completed and submitted for publication in June 2026.

**Conclusions:**

This scoping review will provide the first comprehensive synthesis of AI-, ML-, and DL-based ADHD diagnostic classification studies, mapping the evidence across data modalities, validation practices, interpretability methods, and clinical translation readiness. Findings will inform future methodological standards and support the translation of AI-based diagnostic tools into clinical practice.

## Introduction

### Background

Attention-deficit/hyperactivity disorder (ADHD) is one of the most common neurodevelopmental disorders, affecting over 366 million adults and 139 million children worldwide and constituting a major public health issue. ADHD is characterized by the presence of persistent and developmentally unsuitable symptoms of inattention, hyperactivity, and impulsivity, which affect daily functioning in various domains [1]. It is regarded as the leading psychiatric childhood disorder and has a great negative effect on various aspects of life, such as level of education, employment, and relationships with people [2]. Previous studies provide further evidence that this disorder has a prevalence of approximately 7.6% in children aged between 3 and 12 years and 5.6% in adolescents aged between 12 and 18 years [3]. There is further meta-analytic evidence suggesting that its burden continues far into adulthood—the global prevalence of adult ADHD has been estimated as 3.1%, which is equal to or greater than the prevalence of several other commonly known psychiatric disorders such as bipolar disorder and various anxiety disorders [4]. In addition, the global incidence, prevalence, and disability-adjusted life years of ADHD between 1990 and 2021 rose by 9.9%, 18.7%, and 18.6%, respectively. The groups with the highest disability are children and adolescents [5], pointing to a much-needed change in the field of effective prevention, early diagnosis, and treatment interventions at both the individual and systems levels.

The existing methods of diagnosis of ADHD are based on clinical interviews; behavioral observations; parent and teacher rating scales; and criteria provided in the *Diagnostic and Statistical Manual of Mental Disorders, Fifth Edition*, or the *International Classification of Diseases, 11th Revision* [5]. Such procedures are subjective in nature, time-consuming, and likely to have observer bias, which results in discrepancies in diagnostic accuracy among clinicians and settings [6]. The interrater reliability of ADHD diagnosis is moderate to substantial, which demonstrates a variation in clinical judgment [7]. Additionally, the long period required for the diagnosis of ADHD may lead to delayed treatment, which might cause negative consequences for individuals with ADHD [8].

The limitations of traditional ADHD assessment methods have led researchers to consider objective, data-driven methods. Artificial intelligence (AI), machine learning (ML), and deep learning (DL) offer unprecedented opportunities, enabling researchers to examine intricate, high-dimensional data from many sources, such as neuroimaging (structural and functional magnetic resonance imaging [MRI] and diffusion tensor imaging), electrophysiology (electroencephalography [EEG] and event-related potentials), clinical measures (rating scales and cognitive tests), behavioral measures (motor activity and eye tracking), and digital biomarkers (smartphone use patterns and wearable sensor data) [9,10]. Such AI applications can find nuanced patterns and connections in data that might not be easily observable through traditional clinical assessment, which would provide more precise, efficient, and objective diagnosis of ADHD [11].

During the last 10 years, the number of studies applying AI to ADHD diagnosis has increased greatly. Studies have found that classification accuracies currently lie between 71% and 99%, with especially high performance by EEG-based DL models and clinical assessment–based ML models [12,13]. Nevertheless, even in light of these positive outcomes, there are a number of crucial gaps in the literature. First, studies are highly heterogeneous in terms of study designs, data modalities, sample sizes, and validation methods, so it is challenging to compare their results and evaluate the actual clinical utility of the methods used [14]. Second, the vast majority of studies are based on internal cross-validation or single-site data, and few studies are externally validated on different cohorts, which raises concerns about overfitting and extrapolation to a wide range of populations and clinical settings [15]. Third, there is a lack of standardization in data preprocessing, feature extraction, model design, and evaluation metrics, which hinders reproducibility and comparability [16].

Additionally, most existing studies use binary classification approaches (ADHD vs control) for diagnosis, whereas the effect of different data modalities, feature engineering approaches, and model configurations on diagnostic performance is yet to be investigated [17]. The interpretability and explainability of the models, especially with complex DL models, are not yet well developed, but they are needed to achieve clinical acceptance and trustworthiness [18]. Finally, the evidence base on applications in real-life clinical practice, such as integration with electronic health records, computational needs, cost-effectiveness, and clinical decision-making and patient outcomes, is underdeveloped [19].

Prior review papers have discussed AI in the diagnosis of ADHD, but most of them have involved isolated modalities of data (eg, neuroimaging only or EEG only), have not explicitly covered explainable AI (XAI), have not fully analyzed the quality and generalizability of their datasets, or have not sufficiently discussed the issue of clinical translation [20,21]. Considering the active development of AI technologies and the current literature, a new, more thorough scoping review is required to provide a systematic mapping of the evidence level, determine the strengths and weaknesses of the methodologies, and offer evidence-based suggestions regarding future research and clinical use.

### Rationale for a Scoping Review

The scoping review approach is suitable in the context of our research because the literature on AI-based ADHD diagnosis is heterogeneous in terms of study designs, data types, algorithms, and evaluation methods [22]. In comparison to systematic reviews with a meta-analysis option, which demand homogeneous research with similar results, a scoping review is planned to cover the scope of the evidence and pinpoint important concepts, forms of evidence, and gaps in research [23]. It enables the incorporation of various study designs and methods, offering a holistic view of the field and taking heterogeneity into account, which is not permitted in quantitative syntheses [24].

Considering all these factors, this scoping review will be the most complete of its kind, thoroughly analyzing the entire range of AI, ML, and DL methods used in the diagnosis of ADHD. In addition to the comparison of performance between different data modalities and the analysis of validation strategies, the review will evaluate studies based on their incorporation of model interpretability and explainability. More importantly, the review has a wide geographic and demographic range, with varied age and sex distributions, and represents a broad scan and analysis of the current research landscape. The mapping of the current evidence in this way will allow us to determine the priorities of future research, outline best practices in the methodological sphere, and provide clinically feasible AI-based diagnostic instruments for ADHD.

No current review has, to the best of our knowledge, addressed (1) the entire range of data modalities, from neuroimaging to digital phenotypes; (2) the highly important yet frequently overlooked aspect of model interpretability and explainability; (3) the strict evaluation of external validation practices; and (4) practical routes connecting the accuracy of research to clinical practice. This scoping review fills that gap.

### Review Questions and Objectives

The main aim of the scoping review is to map and synthesize the current research on AI-based classification of ADHD based on articles published after 2019, using rigorous methodological criteria.

The 4 research questions (RQs) that will be addressed in the review are as follows:

- RQ 1: What predictive performance is reported for AI-based ADHD classification, and how is performance evaluated across studies?

This question will examine the spectrum of classification accuracies, sensitivities, specificities, and other performance measures reported in the literature, as well as the validation types used (eg, cross-validation, train-test split, and external validation).

- RQ 2: Which kinds of data (neuroimaging, behavioral data, physiological data, and digital phenotypes) can best be used to assess ADHD using AI?

This question will be used to compare the diagnostic effectiveness of different data modalities and investigate whether multimodal methods are more effective than unimodal methods.

- RQ 3: What dataset characteristics are reported in AI-based ADHD classification studies, and how do studies assess generalizability across cohorts and settings?

This question will explore sample sizes, demographic characteristics, sources of data, and how well the studies have tested the model performance on external or multisite data.

- RQ 4: How can AI models be more interpretable and clinically trustworthy to be used in actual diagnostic processes?

This question will also deal with XAI techniques, feature importance visualization, and techniques to improve the level of transparency and clinical acceptability of models.

### Strengths and Limitations

#### Strengths

This review has several strengths: (1) multimodal coverage that is comprehensive and synthesizes neuroimaging, electrophysiology, clinical testing, behavioral measurements, and digital phenotype evidence sources; (2) a genuine synthesis across multimodal data analysis with respect to all age groups, sex differences, and a wide geographic area; (3) strict PRISMA-ScR (Preferred Reporting Items for Systematic Reviews and Meta-Analyses extension for Scoping Reviews) methodology through manual screening and rigorous inclusion criteria to obtain high-quality evidence; (4) a clear interest in interpretability that covers XAI techniques essential to clinical implementation and credibility; (5) an evidence base that includes recent publications from 2019 to 2026 that have considered recent methodological developments in AI-based diagnosis of ADHD; and (6) a focus on clinical translations based on the gap between the achievements of research and clinical practice.

Even though scoping reviews do not require quality appraisal, we will incorporate a modified version of the Quality Assessment of Diagnostic Accuracy Studies–2 (QUADAS-2) tool to enhance methodological rigor.

#### Limitations

The search is restricted to peer-reviewed publications in English, which might not cover other relevant international and gray literature (such as preprints, conference abstracts, or technical reports). The varieties of study designs, data modalities, and data reporting standards further deter quantitative meta-analysis and potentially limit the richness of comparative synthesis.

## Methods

### Protocol Design and Reporting

This scoping review protocol has been developed based on the Joanna Briggs Institute approach to scoping reviews [25] and the methodological approach by Arksey and O’Malley [24] and improvements by Levac et al [26]. The final scoping review will be presented in accordance with the PRISMA-ScR checklist [27]. This protocol was not preregistered or registered in the Open Science Framework or another protocol registry. This protocol was developed and submitted to *JMIR Research Protocols* in April 2026 after the start of the scoping review, and no separate prospective repository registration was conducted at this time. The review process has since advanced, and most importantly, registering the protocol now would be retrospective and not conducive to receiving the main benefits of preregistration. If there are deviations from this protocol, they will be reported transparently in the final review (see the Changes to the Protocol section).

### Eligibility Criteria

The population, concept, and context framework, which is recommended to be used in scoping reviews in terms of eligibility criteria, was used to define the eligibility criteria for this scoping review [27].

#### Population

Eligible studies should involve participants with a clinical diagnosis of ADHD (confirmed through standardized diagnostic criteria such as the *Diagnostic and Statistical Manual of Mental Disorders, Fifth Edition*, or *International Classification of Diseases, 11th Revision*) compared against non-ADHD control groups (ie, individuals with typical development or without an ADHD diagnosis). Studies involving individuals at risk of ADHD (eg, subclinical or prediagnostic populations) are also eligible provided that the at-risk status is clearly defined by the study authors. Participants may include children, adolescents, adults, or mixed-age samples, with no restrictions on sex, ethnicity, geographic location, or ADHD subtype. Studies must have a total sample size of over 100 participants (ADHD and control groups combined) to be eligible. This threshold was chosen to minimize overfitting and unstable performance estimation, which is well known when using high-dimensional AI, ML, and DL models to train on a very small sample [16,28]. The threshold figures used throughout the literature in the context of the ADHD-200 collection and other multisite repositories, which are the most widely available ADHD benchmark datasets, are far above this threshold, meaning that only very small, single-site studies that are at the highest risk of biased or unstable estimates will not be included. Sample size will not be considered as an indicator of study quality alone but will also be considered during quality assessment (see the Critical Appraisal of Individual Sources of Evidence section) for studies that meet this threshold. This requirement may leave out some smaller but well-designed studies, but it does allow for synthesizing stronger and more interpretable findings.

#### Concept

The concept of interest involves the use of AI, ML, DL, or XAI to diagnose, screen, or classify ADHD. The articles should be empirical primary research papers containing original data and a clear discussion of the ideas inspiring the proposed research. Studies need to clearly state the data modality or dataset the research was based on; the validation or evaluation methodology that was applied; and, at minimum, a single objective test set measure of performance (eg, accuracy, sensitivity, specificity, or area under the curve).

#### Context

Research can be conducted in any environment, such as clinical, research, educational, or community settings. Single-site and multisite studies will be eligible. The studies should be peer-reviewed articles published in journals or conference papers in English between January 2019 and April 2026; the restriction to English-language publications is acknowledged as a limitation as it may exclude relevant studies published in other languages.

#### Exclusion Criteria

Studies will be excluded if they meet any of the following criteria.

AI is applied exclusively to nondiagnostic tasks, such as subtype clustering, prognosis prediction, treatment response prediction, symptom severity estimation, or behavioral monitoring without a primary diagnostic classification component (although the Introduction section identifies these as clinically relevant research gaps, this review focuses specifically on diagnostic classification of ADHD; studies addressing broader applications may be captured in future reviews).

ADHD is not the primary target condition (eg, it is simply listed as a comorbidity or as being in the broad developmental disorder category).

The evaluation design does not allow for the interpretation of participant-level diagnostic performance (eg, data leakage, non–participant-independent validation, or unclear train-test separation).

There is a lack of quality data (eg, extremely small sample sizes, extensive use of synthetic data, or diagnoses made based on untested or self-reported data).

It is not primary research (eg, systematic reviews, scoping reviews, meta-analyses, study protocols without results, editorials, commentaries, and case reports).

It is not a peer-reviewed publication (eg, gray literature, preprints, and technical reports)

The research only involved treatment interventions and did not include diagnostic classification.

### Information Sources and Search Strategy

#### Electronic Databases

An extensive search will be conducted in 5 electronic databases that have been chosen based on their coverage of computer science, engineering, biomedical, and interdisciplinary literature: IEEE Xplore, Scopus, PubMed, Web of Science, and ACM Digital Library.

#### Search Terms and Strategy

The search strategy was developed in consultation with an information specialist and refined through pilot searches to maximize sensitivity and specificity. The 3 clusters of concepts used in the strategy will be mixed with Boolean operators as follows:

- Concept 1 (ADHD)—ADHD OR “Attention-Deficit/Hyperactivity Disorder” OR “Attention Deficit Hyperactivity Disorder”
- Concept 2 (AI technologies)—“Artificial Intelligence” OR “Machine Learning” OR “Deep Learning” OR “Explainable AI” OR “XAI” OR “Explainable Artificial Intelligence” OR “Transformer” OR “Vision Transformer” OR “ViT” OR “Graph Neural Network” OR “GNN” OR “Multimodal” OR “Foundation Model” OR “Large Language Model” OR “LLM” OR “Ensemble Learning” OR “Transfer Learning” OR “Federated Learning” OR “Self-Supervised Learning”
- Concept 3 (diagnostic tasks)—Diagnosis OR Detection OR Classification OR Assessment OR Recognition OR Identification OR Prediction

The search strings will be converted to the syntax requirements of each database and, in the case of databases with a controlled vocabulary (eg, MeSH terms in PubMed), subject headings will be used where necessary. The complete search strategies for all databases can be found in Multimedia Appendix 1. Because the databases differ in indexing and functionality (eg, PubMed supports MeSH controlled vocabulary searching, whereas ACM Digital Library relies more on field-restricted free-text searching), the translated strategies are expected to differ in sensitivity and specificity across databases; to mitigate this, controlled vocabulary will be used where available, and free-text synonyms will be retained across all databases.

#### Search Limits and Filters

Publication date will be from 2019 to 2026, the language of the publications searched will be English, and the publication types will be peer-reviewed journal articles and conference papers.

#### Additional Search Methods

The reference lists of the included studies and other review articles that are relevant but not found in the database searches will be hand searched to identify other eligible studies. Forward citation searching of all included studies will be conducted using Google Scholar to identify additional eligible publications.

#### Search Documentation

Any search strategies will be reported in the final scoping review manuscript; hence, all database-specific syntax, filters, and the number of records recovered will be reported in detail. The search strategy will be peer reviewed by a second information specialist using the Peer Review of Electronic Search Strategies (PRESS) checklist [29].

### Study Selection Process

#### Citation Management

Any records that are retrieved in the database searches will be imported into the EndNote reference management software (Clarivate Analytics). Duplications will be detected and eliminated with the assistance of the automated deduplication function of the EndNote tool, and the process will be further refined through manual scrutiny to find the duplicates that could not be identified automatically (eg, differences in title format and absence of digital object identifiers).

#### Screening Stages

The selection of studies will be conducted in 2 phases.

##### Stage 1: Title and Abstract Screening

The titles and abstracts of all unique records will be screened against the eligibility criteria by 2 independent reviewers. The inclusion and exclusion criteria will be used to create a screening form that will be piloted on 20 records to maintain consistency and to have a common understanding between the reviewers. Any conflicts will be solved through discussion, and in case a consensus cannot be reached, a third reviewer will decide.

##### Stage 2: Full-Text Screening

All records that are considered potentially eligible from title and abstract screening will be retrieved with their full text. Each of the full-text articles will be evaluated by 2 independent reviewers according to the full set of eligibility criteria. A full-text screening form will be used as a standard template, and pilot screening of 5 articles will be conducted to calibrate the reviewers. Conflicts will be settled either through dialogue or arbitration by a third party. Reasons for exclusion at the full-text level will be recorded and reported.

### Interrater Reliability

Interrater agreement will be calculated at both screening stages using the Cohen κ statistic. A κ value of 0.80 or higher will be considered acceptable. If agreement falls below this threshold, reviewers will discuss discrepancies, refine the screening criteria, and conduct additional pilot screening until acceptable agreement is achieved. Three calibration rounds will be conducted at the most, and if the level of agreement is not at the target, screening will be conducted in duplicate, all points not in agreement will be adjudicated by a third reviewer, and the number of rounds will be reported as a limitation of the screening.

### Screening Software

Microsoft Excel will be used for screening with structured forms. Microsoft Excel was selected over dedicated systematic review software (eg, Covidence or Rayyan) due to its flexibility, accessibility to all team members, and compatibility with the data extraction workflow. The reasons behind any screening decisions and the reasons why a study was not selected will be recorded to prevent bias and maintain transparency and repeatability.

### Data Charting Process

#### Data Extraction Form Development

A standardized data extraction form will be developed in Microsoft Excel, aligned with the 4 RQs. The form will be piloted on an initial sample of included studies and iteratively refined based on feedback from the research team and pilot-testing results. In cases in which multiple publications report on overlapping cohorts or datasets, the study with the most complete reporting or the largest sample will be treated as the primary source, and supplementary publications will be flagged to avoid double counting of participants or results in the synthesis. To ensure reproducibility in performance metric extraction, the following rules will be applied: (1) when external validation results are available, these will be prioritized as the primary reported metric; (2) when only internal validation is available, the configuration that the study authors designate as their primary or final model will be recorded, and when a study reports only the best of several internally validated configurations, this will be recorded as such and flagged because reporting the best internal result can introduce optimism and selective reporting bias; and (3) both internal and external results will be extracted and clearly labeled to enable comparison across studies and extractors. The number of candidate models evaluated and whether only the best-performing configuration was reported will also be recorded (Multimedia Appendix 2) so that potential optimism arising from best model selection can be taken into account when interpreting internally validated estimates. Extracted performance estimates will also be stratified by validation tier (independent external validation, held-out internal test set, and cross-validation only) and, within these tiers, specific validation method (eg, k-fold, leave-one-out, nested, or leave-one-site-out cross-validation) will be included as key descriptive variables in the synthesis, and extracted performance estimates will be stratified accordingly. The raw performance will not be compared directly across types of validation because estimates may be more conservative when they are externally validated, and the constraint for comparing raw performance across studies will be reported as a limitation when interpreting the cross-study performance.

#### Pilot-Testing

A sample of 5 included studies will be piloted using the data extraction form by 2 reviewers. The extracted data will be compared by the reviewers, discrepancies will be discussed, and the form and extraction instructions will be refined as required. This will be done repeatedly until an acceptable level of interrater agreement (≥80% agreement on important data items) is attained.

#### Data Extraction Process

The standardized form will be used to extract data from all the studies included by 2 independent reviewers. However, disagreements will be solved through discussion or consultation with a third reviewer. In case of unclear or missing data, the study authors will be contacted to clarify where possible.

### Data Items

The data extraction form and a detailed list of the data items to be extracted per included study, organized by category (bibliographic information; study characteristics; population characteristics; data modality; AI, ML, or DL methods; validation and evaluation; performance metrics; interpretability and explainability; and methodological quality indicators), are provided in Multimedia Appendix 2.

### Critical Appraisal of Individual Sources of Evidence

While critical appraisal of methodological quality is not mandatory for scoping reviews, we will conduct a quality assessment to provide additional context for interpreting findings. A modified version of the QUADAS-2 tool [30] adapted for AI-based diagnostic studies will be used as the AI-specific extension (QUADAS-AI) is still under development. The full modified appraisal form with the AI-specific signaling questions can be found in Multimedia Appendix 3.

Should a validated QUADAS-AI tool become available before the review is completed, we will adopt it and report the change as a protocol amendment. The assessment will focus on 4 domains:

- Data selection: risk of bias pertaining to the selection of the dataset
- Index test: risk of bias concerning the development of AI models and their assessment
- Reference standard: ADHD diagnosis verification risk of bias
- Flow and timing: risk of bias associated with flow and timing of the assessments

Along with the usual QUADAS-2 signaling questions, each domain will contain AI-specific items that address sources of bias specific to ML diagnostic studies. The reviewers will evaluate (1) data leakage (when samples from the same participant are included in the training and test partitions; when features are selected, normalized, or reduced using the entirety of the data prior to partitioning; or when the model includes input variables that will not be available at the time of diagnosis, such as scores of the rating scales used to establish the reference diagnosis or information about ADHD medication use), (2) validation inadequacy (when validation data are not adequate [internal cross-validation, held-out internal test sets, or independent or external validation]), (3) hyperparameter optimization bias (when hyperparameters are tuned based on the test data or when there is no separate validation data partition), (4) feature selection bias (when features are selected outside the cross-validation loop), and (5) treatment of class imbalance and reporting of imbalance-robust metrics in addition to accuracy. All items will be given a rating of low, high, or unclear risk. To standardize the application of each signaling question, a written decision guide with worked examples will be produced, and interreviewer agreement on risk-of-bias judgments will be quantified and reported; both reviewers will pilot the modified tool on a common set of 5 studies and will discuss and resolve discrepancies to ensure consistency between reviewers before moving on.

Quality assessments will be conducted by 2 independent reviewers, and disagreements will be resolved through discussion. No studies will be eliminated based on quality assessment outcomes, and instead, quality rating will be reported descriptively and will be taken into account during findings interpretation.

### Data Synthesis and Presentation of Results

#### Synthesis Approach

Thematic analysis will be used to synthesize data, which will be grouped based on the 4 RQs. Potential sources of heterogeneity are differences in data modality (structural and functional MRI, EEG, clinical and behavioral measures, and digital phenotypes), model families and feature engineering pipelines, ADHD case definitions and reference standards, control group composition, and validation design. Meta-analysis of diagnostic accuracy using bivariate or hierarchical summary receiver operating characteristic models will only be feasible if a sufficient number of studies within a single modality report paired sensitivity and specificity at a common threshold. We anticipate that few studies will report the contingency data required for such pooling and that differences in reference standards and validation design will make any pooled estimate difficult to interpret; we therefore plan a structured narrative synthesis grouped by the 4 RQs rather than quantitative pooling [31].

#### Descriptive Analysis

The characteristics of the studies will be summarized using descriptive statistics, which include (1) number of studies per data modality, (2) distribution of sample sizes, (3) geographic distribution of studies, (4) frequency of algorithm types, (5) frequency of validation strategies, and (6) range and distribution of performance measures.

#### Thematic Synthesis

The results will be integrated into themes as per each RQ.

##### RQ 1 (Performance)

The performance metrics will be summarized in terms of data modality and the type of algorithm. The results that are related to increased classification accuracy will be found and explained, such as the data source and type, the validation strategy, and data quality.

##### RQ 2 (Data Modalities)

The diagnostic value of the various data modalities will be contrasted via trade-offs with respect to cost, accessibility, noise, and performance. Comparison of the added value of multimodal techniques over that of the unimodal approach will be conducted.

##### RQ 3 (Dataset Characteristics)

The characteristics of the dataset will be evaluated with reference to model performance and generalizability. Problems such as the effects of sample size, imbalance in case-control distribution, single-site data vs multisite data, and external validation practices will be discussed.

##### RQ 4 (Interpretability)

The application and kind of XAI practices will be summarized. The degree of the explanations being clinically tested and the probability of their influence on clinical trustworthiness will be addressed.

### Gaps and Future Directions

The synthesis will clearly define gaps in the existing evidence base, limitations of the methodology, and areas of research priorities.

### Piloting and Calibration

Each phase of the review process, such as search strategy, screening, data extraction, and quality assessment, will be piloted to be consistent, reliable, and feasible. Pilot findings (interrater agreement statistics) will be prepared and reported in additional materials.

### Changes to the Protocol

Any form of noncompliance with this protocol when carrying out the scoping review will be recorded justifiably and disclosed openly in the final manuscript.

### Stakeholder Engagement

Various stakeholders in the review process, such as clinicians with a specialty in ADHD, AI researchers and methodologists, and patient and family representatives, will be consulted on the most important development stages or steps of the review process, such as protocol development, preliminary findings analysis, and results interpretation. The clinical relevance of the findings and dissemination strategies will be informed by the input of stakeholders.

The results will be provided in the form of tables, figures, and diagrams and will include (1) a PRISMA-ScR flowchart describing the process of study selection; (2) table summaries of study characteristics; (3) tables of performance measures per modality and algorithm type; (4) figures displaying the trend over time, geographic distribution of studies, and methods used; and (5) network graphs or concept maps illustrating the relationships between the data modalities, algorithms, and results (if applicable).

### Ethical Considerations

This scoping review does not need ethics approval because it entails the synthesis of published information that is publicly available and it does not require the collection of primary data from human participants. As per UK research governance, secondary research that involves the synthesis of information that has already been published in the public realm without the use of human participants, their tissues, or identifiable personal data does not require review by a research ethics committee [32].

## Results

This manuscript reports a protocol, which has not yet been reviewed and has no empirical results as yet. The scoping review began in December 2025. Analysis and screening are in progress, with the scoping review expected to be completed and submitted for publication in June 2026. The review will be finalized with a report on the number of records identified, screened, and included and the characteristics of the included studies. The results will be presented in the form of descriptive statistics (publication patterns and geographical distribution), a thematic synthesis of the results (in the form of the 4 RQs about model performance, data modality contributions, dataset characteristics, and methods of interpreting models), and a visual mapping of the evidence. The study selection process will be presented as a PRISMA-ScR flow diagram (Figure 1), which will be available separately after the searches are conducted.

**Figure 1.**
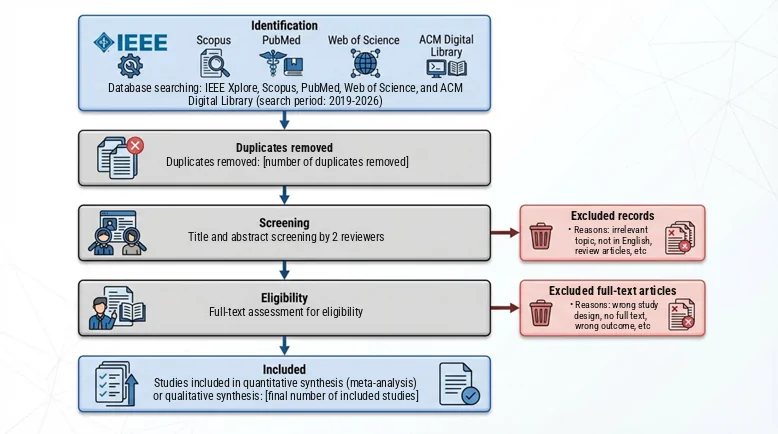
PRISMA-ScR (Preferred Reporting Items for Systematic Reviews and Meta-Analyses extension for Scoping Reviews) flow diagram.

## Discussion

### Principal Findings

Because data collection and screening are ongoing, definitive findings are not yet available. However, given the breadth of evidence identified so far, we expect that this review will feature a fast-growing but still highly methodologically diverse literature with classification accuracy rates that range widely, with few studies externally validated and a steadily increasing number of studies using XAI. We anticipate that the primary challenges to clinical translation will be inconsistent reporting of characteristics of the datasets, class imbalance, and inconsistent validation designs. It is not a lack of accuracy that is expected to be the major problem, since many studies already report high headline accuracy (ie, the best accuracy value among all reported accuracy values highlighted in the abstract), but these values are hard to interpret without appropriate validation.

### Comparison to Prior Work

Several reviews on AI for ADHD diagnosis have appeared recently [20,21,33,34], but most concentrate on a single data modality (eg, MRI or EEG), report headline accuracy without systematically appraising validation rigor or data leakage, and pay limited attention to XAI or to the pathway from research performance to clinical deployment. Recent studies using digital biomarkers and multimodal data further illustrate the field’s shift toward heterogeneous data sources [33,35]. This planned review distinguishes itself from prior work through its coverage of the full range of data modalities, its explicit appraisal of AI-specific sources of bias (including data leakage, feature selection bias, and hyperparameter optimization bias), its emphasis on external validation and interpretability, and its consideration of the broader determinants of clinical translation. Recent EEG-based ML studies continue to report high within-sample classification accuracy while underscoring the persistent need for model interpretability and external validation [36,37].

### Clinical and Research Implications

This review is planned with an explicit focus on interpretability and explainability, which is one of the principal obstacles to the clinical implementation of AI-based diagnostic tools. By charting how primary studies have addressed model transparency and clinical trustworthiness, the review aims to characterize where the field currently stands rather than draw conclusions about clinical effectiveness, which cannot be established before the review has been conducted. Alongside predictive performance, the synthesis is designed to examine the other factors that will influence clinical translation, including regulatory pathways, prospective and external clinical validation, algorithmic fairness and bias among various demographic subgroups, transparency and interpretability, interoperability with clinical information systems, and integration into clinical workflows, as these factors together will shape the viability of AI-driven diagnostic tools in practice.

### Future Research Directions

The review will also identify both methodological assets and flaws in the literature that exists, which will be used to give evidence-based suggestions on how the study designs, reporting practices, and methods of validation can be improved. The review will be useful in directing the allocation of research resources and formulating subsequent studies by identifying gaps in knowledge and what needs to be researched in the future.

In the end, the proposed scoping review can provide insights by filling the knowledge gap between AI literature and clinical practice and fostering the creation of objective, accurate, and trustworthy diagnostic instruments that can positively impact individuals with ADHD.

### Conclusions

This protocol provides a clear and repeatable framework for mapping the research process to diagnose ADHD using AI, focusing on clinical translation, interpretability of models, AI-specific bias, and rigor in validation. The final review should help delineate those aspects of methodology that have been correlated with valid and generalizable performance, as well as providing guidelines for reporting standards in future diagnostic studies using AI.

## Supplementary material

10.2196/99551 Multimedia Appendix 1 Detailed search strategy.

10.2196/99551 Multimedia Appendix 2 Data extraction items.

10.2196/99551 Multimedia Appendix 3 Modified Quality Assessment of Diagnostic Accuracy Studies–2 quality assessment form (adapted for AI-based diagnostic studies).

## Abbreviations

ADHD: attention-deficit/hyperactivity disorder
AI: artificial intelligence
DL: deep learning
EEG: electroencephalography
ML: machine learning
MRI: magnetic resonance imaging
PRESS: Peer Review of Electronic Search Strategies
PRISMA-ScR: Preferred Reporting Items for Systematic Reviews and Meta-Analyses extension for Scoping Reviews
QUADAS-2: Quality Assessment of Diagnostic Accuracy Studies–2
QUADAS-AI: AI-specific extension Quality Assessment of Diagnostic Accuracy Studies
RQ: research question
XAI: explainable AI
