# Supplementary material for: Artificial Intelligence, Machine Learning, and Deep Learning Approaches for Attention-Deficit/Hyperactivity Disorder Diagnosis: Protocol for a Scoping Review
Source: JMIR Res Protoc. 2026 Oct 7;15:e99551. doi: 10.2196/99551 (PMC13645319; doi:10.2196/99551)
Supplement: Supplementary file 1 [file resprot-v15-e99551-s001.docx]

# Appendix 1: Detailed Search Strategy

*IEEE Xplore Search Strategy*

**Search string:**

((("All Metadata":"ADHD") OR ("All Metadata":"Attention-Deficit/Hyperactivity Disorder")) AND (("All Metadata":"Artificial Intelligence") OR ("All Metadata":"Machine Learning") OR ("All Metadata":"Deep Learning") OR ("All Metadata":"Explainable AI") OR ("All Metadata":"XAI") OR ("All Metadata":"Transformer") OR ("All Metadata":"Vision Transformer") OR ("All Metadata":"Graph Neural Network") OR ("All Metadata":"Multimodal") OR ("All Metadata":"Foundation Model") OR ("All Metadata":"Large Language Model") OR ("All Metadata":"Ensemble Learning") OR ("All Metadata":"Transfer Learning") OR ("All Metadata":"Federated Learning") OR ("All Metadata":"Self-Supervised Learning")) AND (("All Metadata":"Diagnosis") OR ("All Metadata":"Detection") OR ("All Metadata":"Classification") OR ("All Metadata":"Assessment") OR ("All Metadata":"Recognition") OR ("All Metadata":"Identification") OR ("All Metadata":"Prediction")))

**Filters:** - Publication Years: 2019–2026 - Article Type: Journals and Conference Proceedings - Language: English

*Scopus Search Strategy*

**Search string:**

( TITLE-ABS-KEY ( ADHD ) OR TITLE-ABS-KEY ( "Attention-Deficit/Hyperactivity Disorder" ) ) AND ( TITLE-ABS-KEY ( "Artificial Intelligence" ) OR TITLE-ABS-KEY ( "Machine Learning" ) OR TITLE-ABS-KEY ( "Deep Learning" ) OR TITLE-ABS-KEY ( "Explainable AI" ) OR TITLE-ABS-KEY ( XAI ) OR TITLE-ABS-KEY ( Transformer ) OR TITLE-ABS-KEY ( "Vision Transformer" ) OR TITLE-ABS-KEY ( "Graph Neural Network" ) OR TITLE-ABS-KEY ( Multimodal ) OR TITLE-ABS-KEY ( "Foundation Model" ) OR TITLE-ABS-KEY ( "Large Language Model" ) OR TITLE-ABS-KEY ( "Ensemble Learning" ) OR TITLE-ABS-KEY ( "Transfer Learning" ) OR TITLE-ABS-KEY ( "Federated Learning" ) OR TITLE-ABS-KEY ( "Self-Supervised Learning" ) ) AND ( TITLE-ABS-KEY ( Diagnosis ) OR TITLE-ABS-KEY ( Detection ) OR TITLE-ABS-KEY ( Classification ) OR TITLE-ABS-KEY ( Assessment ) OR TITLE-ABS-KEY ( Recognition ) OR TITLE-ABS-KEY ( Identification ) OR TITLE-ABS-KEY ( Prediction ) ) AND PUBYEAR > 2018 AND PUBYEAR < 2027 AND ( LIMIT-TO ( SRCTYPE , "j" ) OR LIMIT-TO ( SRCTYPE , "p" ) ) AND ( LIMIT-TO ( DOCTYPE , "ar" ) OR LIMIT-TO ( DOCTYPE , "cp" ) OR LIMIT-TO ( DOCTYPE , "re" ) OR LIMIT-TO ( DOCTYPE , "cr" ) ) AND ( LIMIT-TO ( LANGUAGE , "English" ) )

**Filters:** - Publication Years: 2019–2026 - Article Type: Journals and Conference Proceedings - Language: English

*PubMed Search Strategy*

**Search string:**

(("Attention Deficit Disorder with Hyperactivity"[MeSH Terms] OR ADHD[tiab] OR "Attention-Deficit/Hyperactivity Disorder"[tiab]) AND ("Artificial Intelligence"[MeSH Terms] OR "Machine Learning"[MeSH Terms] OR "Deep Learning"[MeSH Terms] OR "Artificial Intelligence"[tiab] OR "Machine Learning"[tiab] OR "Deep Learning"[tiab] OR "Explainable AI"[tiab] OR XAI[tiab] OR Transformer[tiab] OR "Vision Transformer"[tiab] OR "Graph Neural Network"[tiab] OR Multimodal[tiab] OR "Foundation Model"[tiab] OR "Large Language Model"[tiab] OR "Ensemble Learning"[tiab] OR "Transfer Learning"[tiab] OR "Federated Learning"[tiab] OR "Self-Supervised Learning"[tiab]) AND ("Diagnosis"[MeSH Terms] OR "Classification"[MeSH Terms] OR Diagnosis[tiab] OR Detection[tiab] OR Classification[tiab] OR Assessment[tiab] OR Recognition[tiab] OR Identification[tiab] OR Prediction[tiab]))

**Filters:** - Publication Years: 2019–2026 - Article Type: Journals and Conference Proceedings - Language: English

*Web of Science Search Strategy*

**Search string:**

TS=(ADHD OR "Attention-Deficit/Hyperactivity Disorder") AND TS=("Artificial Intelligence" OR "Machine Learning" OR "Deep Learning" OR "Explainable AI" OR XAI OR "Explainable Artificial Intelligence" OR "Transformer" OR "Vision Transformer" OR "Graph Neural Network" OR "Multimodal" OR "Foundation Model" OR "Large Language Model" OR "Ensemble Learning" OR "Transfer Learning" OR "Federated Learning" OR "Self-Supervised Learning") AND TS=(diagnos* OR detect* OR classif* OR assess* OR recogni* OR identif* OR predict*)

**Filters:** - Publication Years: 2019–2026 - Article Type: Journals and Conference Proceedings - Language: English

*ACM Digital Library Search Strategy*

**Search string:**

(Abstract:("ADHD" OR "Attention-Deficit/Hyperactivity Disorder") OR Keyword:("ADHD" OR "Attention-Deficit/Hyperactivity Disorder") OR Title:("ADHD" OR "Attention-Deficit/Hyperactivity Disorder")) AND (Abstract:("Artificial Intelligence" OR "Machine Learning" OR "Deep Learning" OR "Explainable AI" OR "XAI" OR "Transformer" OR "Vision Transformer" OR "Graph Neural Network" OR "Multimodal" OR "Foundation Model" OR "Large Language Model" OR "Ensemble Learning" OR "Transfer Learning" OR "Federated Learning" OR "Self-Supervised Learning") OR Keyword:("Artificial Intelligence" OR "Machine Learning" OR "Deep Learning" OR "Explainable AI" OR "XAI" OR "Transformer" OR "Vision Transformer" OR "Graph Neural Network" OR "Multimodal" OR "Foundation Model" OR "Large Language Model" OR "Ensemble Learning" OR "Transfer Learning" OR "Federated Learning" OR "Self-Supervised Learning") OR Title:("Artificial Intelligence" OR "Machine Learning" OR "Deep Learning" OR "Explainable AI" OR "XAI" OR "Transformer" OR "Vision Transformer" OR "Graph Neural Network" OR "Multimodal" OR "Foundation Model" OR "Large Language Model" OR "Ensemble Learning" OR "Transfer Learning" OR "Federated Learning" OR "Self-Supervised Learning")) AND (Abstract:("Diagnosis" OR "Detection" OR "Classification" OR "Assessment" OR "Recognition" OR "Identification" OR "Prediction") OR Keyword:("Diagnosis" OR "Detection" OR "Classification" OR "Assessment" OR "Recognition" OR "Identification" OR "Prediction") OR Title:("Diagnosis" OR "Detection" OR "Classification" OR "Assessment" OR "Recognition" OR "Identification" OR "Prediction"))

**Filters:** - Publication Years: 2019–2026 - Article Type: Journals and Conference Proceedings - Language: English
