# Supplementary material for: Artificial Intelligence, Machine Learning, and Deep Learning Approaches for Attention-Deficit/Hyperactivity Disorder Diagnosis: Protocol for a Scoping Review
Source: JMIR Res Protoc. 2026 Oct 7;15:e99551. doi: 10.2196/99551 (PMC13645319; doi:10.2196/99551)
Supplement: Supplementary file 2 [file resprot-v15-e99551-s002.docx]

# Appendix 2: Data Extraction

[Detailed Excel-based extraction form with all variables listed in the Data Items section, including dropdown menus for categorical variables, validation rules for numerical variables, and instructions for each field]

**Key Sections:** 1. Study Identification 2. Bibliographic Information 3. Study Characteristics 4. Population Characteristics 5. Data Modality 6. AI/ML/DL Methods 7. Validation and Evaluation 8. Performance Metrics 9. Interpretability and Explainability 10. Methodological Quality Indicators 11. Extractor Notes.

## Data Items

The full set of data items to be extracted from each included study is listed below, organised by category. These items correspond to the fields captured in the extraction form described above.

### Bibliographic Information

- Authors
- Publication year
- Journal or conference name
- Country of corresponding author
- Funding source

### Study Characteristics

- Study objectives
- Study design
- Setting (clinical, research, educational, community)
- Geographic location

### Population Characteristics

- Total sample size
- ADHD group sample size
- Control group sample size
- Age range and mean age
- Sex distribution
- ADHD subtype(s) included
- Comorbidities reported
- Diagnostic criteria used (DSM-5, ICD-11, other)
- Recruitment source

### Data Modality

- Primary data type (neuroimaging, electrophysiology, clinical assessment, behavioral, digital phenotype, multimodal)
- Specific modality details (e.g., fMRI, EEG, rating scales, eye tracking)
- Dataset name (if publicly available dataset used)
- Data collection procedures

### AI/ML/DL Methods

- Algorithm type (e.g., support vector machine, random forest, convolutional neural network, recurrent neural network)
- Feature engineering approach
- Dimensionality reduction methods
- Hyperparameter optimization strategy

### Validation and Evaluation

- Validation strategy (k-fold cross-validation, train-test split, external validation)
- Number of folds (if cross-validation)
- Train-test split ratio (if applicable)
- Use of independent test set
- Multi-site validation (yes/no)

### Performance Metrics Reported

- Accuracy
- Sensitivity (recall)
- Specificity
- Precision
- F1-score
- Area under the receiver operating characteristic curve (AUC-ROC)
- Other metrics reported

### Interpretability and Explainability

- Use of explainable AI methods (yes/no)
- Specific XAI techniques employed (e.g., SHAP, Grad-CAM, attention mechanisms, feature importance)
- Clinical validation of explanations (yes/no)

### Methodological Quality Indicators

- Risk of data leakage
- Number of candidate models or architectures evaluated
- Whether only the best-performing model or configuration was reported (yes/no)
- Class balance and ADHD prevalence within the study sample (ADHD-to-control ratio)
- Handling of class imbalance
- Control for confounding variables
- Reporting completeness
