# Supplementary material for: Artificial Intelligence, Machine Learning, and Deep Learning Approaches for Attention-Deficit/Hyperactivity Disorder Diagnosis: Protocol for a Scoping Review
Source: JMIR Res Protoc. 2026 Oct 7;15:e99551. doi: 10.2196/99551 (PMC13645319; doi:10.2196/99551)
Supplement: Supplementary file 3 [file resprot-v15-e99551-s003.docx]

# Appendix 3: Modified QUADAS-2 Quality Assessment Form (Adapted for AI-Based Diagnostic Studies)

In QUADAS-2, **each domain is evaluated using two major judgements [30]:**

1. **Risk of Bias** → *Main methodological validity question*
1. **Applicability Concerns** → *Relevance to your review question*

### Modified QUADAS-2 for AI-Based Diagnostic Studies

**Study ID:** ______________________________

**Reviewer:** ______________________________

**Date:** ______________________________

**Domain 1: Patient Selection**

*Risk of Bias* - Was a consecutive or random sample of patients enrolled? ☐ Yes ☐ No ☐ Unclear

Was a case-control design avoided? ☐ Yes ☐ No ☐ Unclear

Did the study avoid inappropriate exclusions? ☐ Yes ☐ No ☐ Unclear

**Risk of Bias:** ☐ Low ☐ High ☐ Unclear

*Applicability* - Are there concerns that the included patients and setting do not match the review question? ☐ Low ☐ High ☐ Unclear

**Domain 2: Index Test (AI/ML/DL Model)**

*Risk of Bias* - Were the AI model development and validation procedures adequately described? ☐ Yes ☐ No ☐ Unclear

Was the AI model evaluated without knowledge of the reference standard results? ☐ Yes ☐ No ☐ Unclear

Was overfitting avoided (e.g., through appropriate train-test split, cross-validation, external validation)? ☐ Yes ☐ No ☐ Unclear

Was subject-level independence between training and test data maintained, with no data leakage? ☐ Yes ☐ No ☐ Unclear

Were feature selection, normalisation, and dimensionality reduction performed only within training folds (no information leakage)? ☐ Yes ☐ No ☐ Unclear

Was hyperparameter tuning performed using a validation partition separate from the test set? ☐ Yes ☐ No ☐ Unclear

Was the model validated on an independent or external dataset? ☐ Yes ☐ No ☐ Unclear

Was class imbalance addressed, and were imbalance-robust metrics (e.g., sensitivity, specificity, AUC) reported alongside accuracy? ☐ Yes ☐ No ☐ Unclear

**Risk of Bias:** ☐ Low ☐ High ☐ Unclear

*Applicability* - Are there concerns that the AI model, its conduct, or interpretation differ from the review question? ☐ Low ☐ High ☐ Unclear

**Domain 3: Reference Standard**

*Risk of Bias* - Is the reference standard (ADHD diagnosis) likely to correctly classify the target condition? ☐ Yes ☐ No ☐ Unclear

Were the reference standard results interpreted without knowledge of the AI model results? ☐ Yes ☐ No ☐ Unclear

**Risk of Bias:** ☐ Low ☐ High ☐ Unclear

*Applicability* - Are there concerns that the target condition as defined by the reference standard does not match the review question? ☐ Low ☐ High ☐ Unclear

**Domain 4: Flow and Timing**

*Risk of Bias* - Was there an appropriate interval between the AI model evaluation and reference standard? ☐ Yes ☐ No ☐ Unclear

Did all patients receive the same reference standard? ☐ Yes ☐ No ☐ Unclear

Were all patients included in the analysis? ☐ Yes ☐ No ☐ Unclear

**Risk of Bias:** ☐ Low ☐ High ☐ Unclear

**Overall Quality Assessment:** ☐ Low Risk ☐ Some Concerns ☐ High Risk
